# Integrated proteomic analysis of low-grade gliomas reveals contributions of 1p-19q co-deletion to oligodendroglioma

**DOI:** 10.1186/s40478-022-01372-1

**Published:** 2022-05-07

**Authors:** Derek Wong, Tae Hoon Lee, Amy Lum, Valerie Lan Tao, Stephen Yip

**Affiliations:** 1 Pathology and Laboratory Medicine, Vancouver General Hospital, University of British Columbia, Vancouver, Canada; 2 Molecular Oncology, BC Cancer Agency, Vancouver, Canada

**Keywords:** Low grade glioma, Oligodendroglioma, Astrocytoma, IDH wildtype, CIC mutation, 1p19q codeletion, Proteomics, FFPE, Genomics, Transcriptomics

## Abstract

Diffusely infiltrative low-grade gliomas (LGG) are primary brain tumours that arise predominantly in the cerebral hemispheres of younger adults. LGG can display either astrocytic or oligodendroglial histology and do not express malignant histological features. Vast majority of LGG are unified by IDH mutations. Other genomic features including ATRX as well as copy number status of chromosomes 1p and 19q serve to molecularly segregate this tumor group. Despite the exponential gains in molecular profiling and understanding of LGG, survival rates and treatment options have stagnated over the past few decades with few advancements. In this study, we utilize low grade glioma RNA-seq data from the Cancer Genome Atlas (TCGA-LGG) and tandem mass-spectrometry on an in-house cohort of 54 formalin-fixed paraffin-embedded (FFPE) LGG specimens to investigate the transcriptomic and proteomic profiles across the three molecular subtypes of LGG (Type I: IDH mutant – 1p19q co-deleted, Type II: IDH mutant – 1p19q retained, Type III: IDH wildtype). Within the 3 LGG subtypes, gene expression was driven heavily by IDH mutation and 1p19q co-deletion. In concordance with RNA expression, we were able to identify decreased expressions of proteins coded in 1p19q in Type I LGG. Further proteomic analysis identified 54 subtype specific proteins that were used to classify the three subtypes using a multinomial regression model (AUC = 0.911). Type I LGG were found to have increased protein expression of several metabolic proteins while Type III LGG were found to have increased immune infiltration and inflammation related proteins. Here we present the largest proteomic cohort of LGG and show that proteomic profiles can be successfully analyzed from FFPE tissues. We uncover previously known and novel subtype specific markers that are useful for the proteomic classification of LGG subtypes.

**Supplementary Information:**

The online version contains supplementary material available at 10.1186/s40478-022-01372-1.

## Introduction

Diffusely infiltrative Low grade glioma (LGG) are primary tumours of the brain classified as grade 2/3 neoplasms by the World Health Organization (WHO) and arise primarily in the cerebral hemispheres of younger adults [1]. Despite the exponential gains in molecular profiling and understanding of LGG, survival rates and treatment options have stagnated over the past few decades with few advancements [2]. With the 2021 update to the WHO classification of tumours of the central nervous system, the use of molecular data, namely IDH mutation and whole arm codeletion of chromosomes 1p and 19q, now supersedes classical histology based classification of LGG [1]. Using these molecular markers, LGGs can now be stratified into three molecularly distinct prognostic subgroups. Oligodendroglioma (ODG) and astrocytoma are both classified by mutations in *IDH1* or *IDH2* (IDH) with the former also harboring 1p19q codeletion and the later retaining 1p19q [3]. ODG are also associated with *CIC* mutations (up to 70%) [4]^5^, while IDH- mutant astrocytoma frequently harbour mutations in *TP53* and *ATRX* [6]. The remaining tumours which lack IDH mutations are termed IDH wildtype glioblastoma (GBM) and are most often associated with *TERT* promoter mutations, gains of chromosome 7/*EGFR*, and loss of chromosome 10, characteristic of high grade (grade 4) GBM [7].

Current treatment for LGG varies depending on the molecular subtype, grade, and location/resection and can include clinical monitoring, chemotherapy (procarbazine/CCNU/vincristine or PCV and temozolomide), and radiotherapy. Clinically, ODG tumours respond well to radio- and chemotherapy and are associated with the best prognosis [8]^9^. Conversely, IDH wildtype tumours, even in the absence of high grade histology, are associated with the worst prognosis and IDH mutant astrocytoma are associated with a variable but intermediate response [10]^11^. A subset of these tumours, regardless of molecular subtype, will progress towards high grade GBM and death.

While our understanding of LGG biology has made tremendous progress, the vast majority of these discoveries have been within the genomic [3], transcriptomic [12, 13], and epigenomic [14, 15] space with little exploration in the proteomic landscape of LGG. Recent advances in proteomic profiling capabilities have enabled to use of formalin fixed paraffin embedded (FFPE) tissues which greatly increase the ease of sample curation and may facilitate further insights into the drivers of response within the 3 subtypes [16, 17],^18^. In GBM, proteomic profiling has demonstrated the ability to stratify patient survival independent of transcriptomic and pathway signatures [19–21][22]. Discordant transcriptome and proteome suggest that proteomic studies may provide advantages for the discovery of actionable targets that translate into clinical and immunohistochemical validation.

In this study, we utilise low grade glioma RNA-seq data from the Cancer Genome Atlas (TCGA-LGG) to investigate the transcriptomic profiles across the three molecular subtypes of LGG and find that IDH mutation and 1p19q co-deletion drive genome wide transcriptomic profiles. Interestingly, while *CIC* mutations within ODGs were associated with increased receptor tyrosine kinase (RTK) activation, it did not result in robust differential clustering on a global transcriptomic level. We further explore the proteomic landscape of LGG by performing tandem mass-tagged mass spectrometry on a cohort of in-house genomically characterized FFPE LGG. Proteogenomic analysis uncovered previously identified and novel protein biomarkers in LGG which were used to build a subtype classifier.

## Materials and methods

### Patient cohort

Formalin fixed paraffin embedded and fresh frozen tumour samples analyzed were from 108 adults with previously untreated LGG (WHO grades 2/3), including 45 oligodendrogliomas, 45 astrocytomas, and 18 glioblastomas from Vancouver General Hospital. Diagnoses were established from routine neuropathological and molecular workup at Vancouver General Hospital and reviewed by a neuropathologist for this study. Patient cohorts are described in Additional file 6: Table S1. This study was approved by the institutional review board (H08-2838) and informed written consent was obtained from all patients.

### Targeted DNA panel sequencing

Genomic DNA was extracted using the AllPrep DNA/RNA FFPE kit (Qiagen) or AllPrep DNA/RNA kit (Qiagen) depending on sample type (FFPE or snap frozen). Custom DNA panel was designed on the Illumina DesignStudio (Illumina) using hg19 as the reference genome. Panel design can be found in Additional file 7: Table S2. Library preparation was performed using Ampliseq for Illumina On-Demand, Custom and Community panels according to manufacturer’s protocol. Sequencing was performed on an Illumina MiSeq with using a 600 cycle (v3) kit with Paried End 150 bp reads with an average depth of 1371.84X (Additional file 8: Table S3). Assembly was estimated using Cufflinks (http://cole-trapnelllab.github.io/cufflinks/) through bioinformatics apps available on Illumina Sequence Hub [23]. Single nucleotide variants were detected using Mutect (v1.1.5) [24]. Insertions and Deletions were detected using Strelka (v2.9.9) [25]. Copy number variations for chromosomes 1p and 19q were called using OncoCNV (v.1.2.0) [26].

### Tissue lysis and enzymatic digestion for proteomic analysis

Tissue processing was carried out as described previously [16]. FFPE tissue Sects. (2 × 10 µm scrolls) were provided on glass slides for processing. Tissue was scraped and suspended with lysis buffer (100 mM HEPES pH 8 [H3375, Sigma], 4% SDS [L6026, Sigma], 10 mM TCEP [C4706, Sigma], 40 mM CAA [C0267, Sigma], and 1 × complete protease inhibitor – EDTA free [4693159001, Sigma]). Mixtures were heated at 90 °C for 90 min, and chilled to room temperature for 15 min. Protein from flash frozen tissue samples were extracted using AllPrep DNA/RNA/Protein Mini Kit (80,004, Qiagen). Prior to digestion, samples were cleaned using a variation on the SP3 protocol [17]. Briefly, to each protein mixture, 200 μg of SP3 beads was added and mixed. To induce protein binding to the beads, 100% by volume of acetonitrile was added per sample. Bead-protein solutions were mixed and incubated for a total of 10 min at room temperature then placed on a magnetic rack for 2 min and the supernatant discarded. The beads were rinsed twice with 180μL of freshly prepared 70% ethanol and once with 180μL of 100% ethanol. Rinsed beads were reconstituted in aqueous buffer (~ 50μL, 0.2 M HEPES pH 8) containing a 1:50 (μg:μg) enzyme to protein amount of trypsin/LysC mix (Promega, CAT#V5071), and briefly sonicated in a water bath (30 s) to disaggregate the beads. Mixtures were incubated for 14 h at 37 °C in a PCR thermocycler then sonicated briefly (10 s) in a water bath to resuspend the beads. The supernatants were recovered using a magnetic rack and transferred to fresh 1.5 mL polypropylene micro-tubes.

### TMT labeling

Prior to labeling, TMT labels were removed from the −80 °C freezer and allowed to equilibrate at room temperature. TMT label was added in two volumetrically equal steps to achieve a 2:1 (μg:μg) TMT label to peptide final concentration, 30 min apart. All incubations were carried out at room temperature. Reactions were quenched with glycine. Labeled peptides were concentrated in a SpeedVac centrifuge, combined, and run through a SepPak cartridge for clean-up prior to HPLC fractionation.

### HPLC fractionation

High-pH reversed phase analysis was performed on an Agilent 1100 HPLC system equipped with a diode array detector (254, 260, and 280 nm). Fractionation was performed on a Kinetix EVO C18 column (2.1 × 150 mm, 1.7 μm core shell, 100 Å, Phenomenex). Elution was performed at a flow rate of 0.2 mL per minute using a gradient of mobile phase A (10 mM ammonium bicarbonate, pH 8) and B (acetonitrile), from 3 to 35% over 60 min. Fractions were collected every minute across the elution window for a total of 48 fractions, which were concatenated to a final set of 12 (e.g. 1 + 13 + 25 + 37 = fraction 1). Fractions were dried in a SpeedVac centrifuge and reconstituted in 1% formic acid with 1% DMSO in water prior to MS analysis.

### Mass spectrometry analysis

Analysis of TMT labeled peptide fractions was carried out on an Orbitrap Fusion Tribrid MS platform (Thermo Scientific). Samples were introduced using an Easy-nLC 1000 system (Thermo Scientific). Columns used for trapping and separations were packed in-house. Trapping columns were packed in 100 μm internal diameter capillaries to a length of 25 mm with C18 beads (Reprosil-Pur, Dr. Maisch, 3 μm particle size). Trapping was carried out for a total volume of 10 μL at a pressure of 400 bar. After trapping, gradient elution of peptides was performed on a C18 (Reprosil-Pur, Dr. Maisch, 1.9 μm particle size) column packed in-house to a length of 15 cm in 100 μm internal diameter capillaries with a laser-pulled electrospray tip and heated to 45 °C using AgileSLEEVE column ovens (Analytical Sales & Service). Elution was performed with a gradient of mobile phase A (water and 0.1% formic acid) and B (acetonitrile and 0.1% formic acid) over 120-min at a flow rate of 300nL/min. Data acquisition on the Orbitrap Fusion (control software version 2.1.1565.20) was carried out using a data-dependent method with multi-notch synchronous precursor selection MS3 scanning for TMT tags. Survey scans covering the mass range of 350 – 1500 were acquired at a resolution of 120,000 (at m/z 200), with quadrupole isolation enabled, an S-Lens RF Level of 60%, a maximum fill time of 50 ms, and an automatic gain control (AGC) target value of 5e5. For MS2 scan triggering, monoisotopic precursor selection was enabled, charge state filtering was limited to 2 – 4, an intensity threshold of 5e3 was employed, and dynamic exclusion of previously selected masses was enabled for 60 s with a tolerance of 20 ppm. MS2 scans were acquired in the ion trap in Rapid mode after CID fragmentation with a maximum fill time of 150 ms, quadrupole isolation, an isolation window of 1 m/z, collision energy of 30%, activation Q of 0.25, injection for all available parallelizable time turned OFF, and an AGC target value of 4e3. Fragment ions were selected for MS3 scans based on a precursor selection range of 400-1200 m/z, ion exclusion of 20 m/z low and 5 m/z high, and isobaric tag loss exclusion for TMT. The top 10 precursors were selected for MS3 scans that were acquired in the Orbitrap after HCD fragmentation (NCE 60%) with a maximum fill time of 150 ms, 50,000 resolution, 110–750 m/z scan range, ion injection for all parallelizable time turned OFF, and an AGC target value of 1e5. The total allowable cycle time was set to 4 s. MS1 and MS3 scans were acquired in profile mode, and MS2 in centroid format.

### Mass spectrometry data analysis

Data from the Orbitrap Fusion were processed using Proteome Discoverer Software (ver. 2.1.1.21) [27]. MS2 spectra were searched using Sequest HT against a combined UniProt Human proteome database appended to a list of common contaminants (24,624 total sequences). Sequest HT parameters were specified as: trypsin enzyme, 2 missed cleavages allowed, minimum peptide length of 6, precursor mass tolerance of 20 ppm, and a fragment mass tolerance of 0.6. Oxidation of methionine, and TMT at lysine and peptide N-termini were set as variable modifications. Carbamidomethylation of cysteine was set as a fixed modification. Peptide spectral match error rates were determined using the target-decoy strategy coupled to Percolator modeling of positive and false distributions [27, 28]. Data were filtered at the peptide spectral match-level to control for false discoveries using a q-value cut-off of 0.01 as determined by Percolator. Data sets generated in Proteome Discoverer were exported and analyzed with a combination of scripts built in R designed in-house. Contaminant and decoy proteins were removed from all data sets prior to downstream analysis.

### Transcriptomic analyses

RNA-sequencing results were obtained from TCGA using http://firebrowse.org/doi:10.7908/C11G0KM9 and mutation and copy number data were obtained from TCGA using http://www.cbioportal.org/ [29]. DEA was performed using the R package DEseq2 [30].

### Gene set enrichment analysis

The Metascape software https:// metascape.org was used to perform functional enrichments using the multiple gene lists mode [31]. Gene ontology (GO) Biological Processes, Hallmark Gene Sets and Oncogenic Signatures were used for enrichment analyses of all DE genes, with a p-value cut-off of 0.05, and a minimum enrichment of 1.5. Only terms with a BH-adjusted p-value < 0.05 were retained.

## Results

### LGG transcriptome driven by IDH mutation and 1p/19q co-deletion

**Fig. 1:**
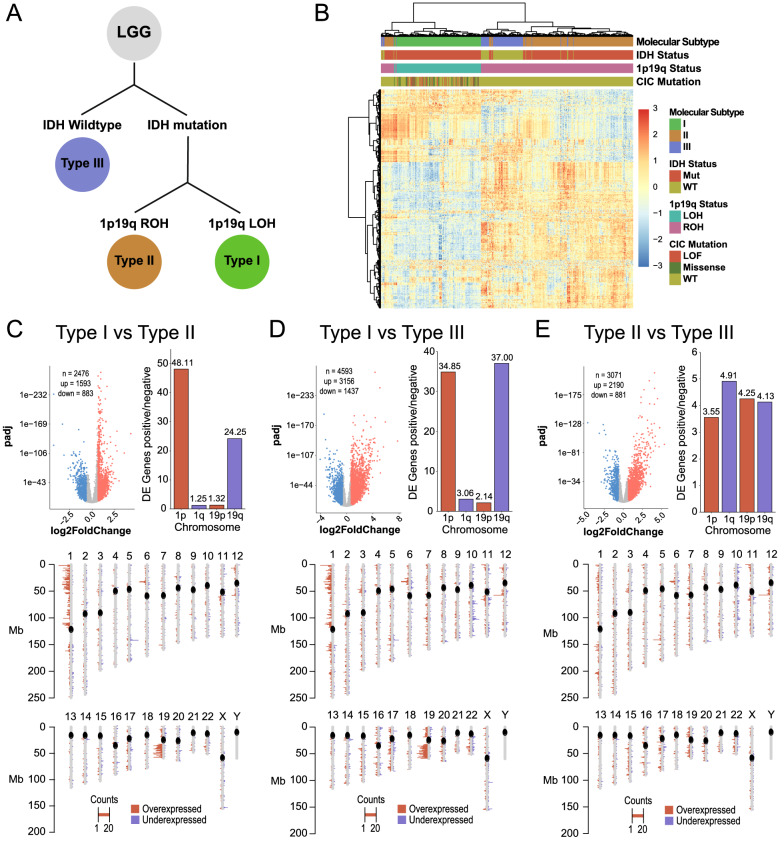
**A** Schematic for classification of LGG subtypes based upon molecular information. **B** Unsupervised clustering and heatmap of TCGA LGG samples based upon molecular information. **C**-**E** Left: Volcano plot of differentially expressed genes. Right: Ratio of number of up and down regulated genes identified on chromosomes 1p/q and 19p/q. Bottom: Chromplot of the locations of up and downregulated genes identified through differential expression analysis

To investigate the transcriptome profiles of LGG, we utilised the publicly available mRNA-seq data from the TCGA lower grade glioma (TCGA-LGG) data set and annotated samples based upon the status of IDH and 1p19q. IDH- mutant/1p19q- codeleted LGG were labeled as Type I (n = 91), IDH- mutant/1p19q- retained LGG were termed as Type II (n = 100), and IDH- wildtype LGG were labeled as Type III (n = 54; Fig. 1A). TCGA-LGG samples segregated into their respective molecular subgroups using unsupervised clustering of the top 500 variably expressed (median absolute deviation) genes (Fig. 1B). Differential expression analysis (DEA) between the three molecular subgroups identified 2476 (I vs II), 4593 (I vs III), and 3071 (II vs III) differentially expressed (DE) genes (Fold change > 1.5, padj < 0.01; Additional file 9: Table S4). When factoring the location of each DE gene, we observed an enrichment of DE genes located on 1p and 19q in Type I LGG suggesting that the hallmark 1p19q co-deletion drives its transcriptomic signature (Fig. 1C and D). This enrichment was not observed between Type II and Type III LGG (Fig. 1E). Gene set enrichment analysis (GSEA) between the three molecular subgroups revealed upregulation of neuronal and glial differentiation signatures in Type I, cytoskeletal morphogenesis in Type II, and angiogenesis, receptor tyrosine kinase (RTK) signaling, and inflammatory signatures in Type III (Additional file 1: Fig S1, Additional file 10: Table S5).

### Type I LGG *CIC* mutations do not result in distinct transcriptomic signatures

Studies have shown Type I LGGs with loss of function or missense *CIC* mutations exhibit dysregulation of pathways involved in RTK signaling [32–34]. Interestingly, while *CIC* mutations have not been found to correlate with survival [35], loss of CIC protein expression has been correlated with decreased survival [36]. This may suggest that Type I LGG may be stratified based upon CIC protein status and may explain the lack of *CIC*-transcriptome- driven clustering within Type I LGG observed in Fig. 1B. Few studies have also investigated the differences between *CIC* loss of function and missense mutations. *CIC* missense mutations most often occur in exon 5 which contains the DNA binding domain, or exon 19 which contains the C1-motif responsible for stabilizing CIC-DNA interaction [37]. To investigate this further, unsupervised clustering was performed using only Type I LGG samples (top 500) which did not result in *CIC*-status dependent clustering (Additional file 2: Fig. S2).

**Fig. 2:**
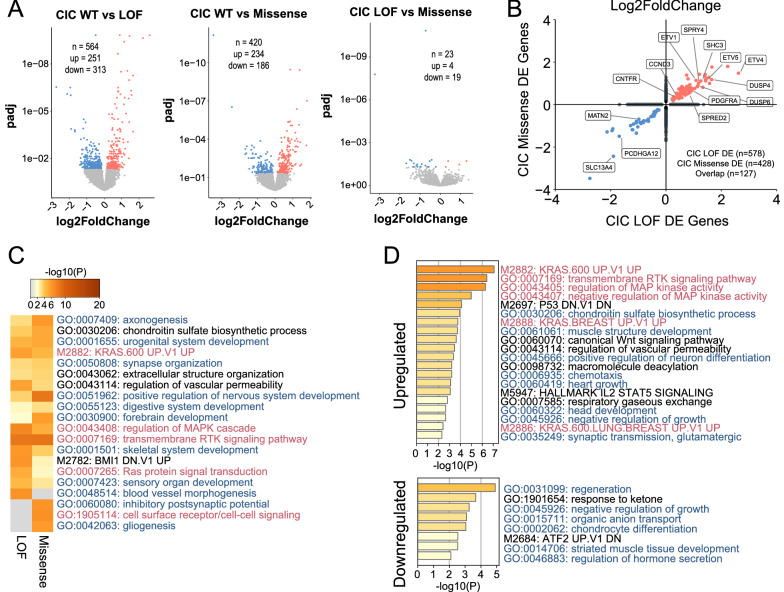
**A** Volcano plots showing differentially expressed genes identified between TCGA *CIC* mutation groups within Type I LGG. **B** Comparison of expression between differentially expressed genes identified in *CIC* LOF and *CIC* missense Type I LGGs **C** Heatmap of gene set enrichment analysis comparison between *CIC* LOF and *CIC* missense Type I LGG D) Gene set enrichment analysis for up (top) and down (bottom) regulated differentially expressed genes shared between *CIC* LOF and *CIC* missense Type I LGG. * Gene sets in red related to MAPK/RTK and blue related to neuronal/development/differentiation

Within Type I LGG, we further subdivided these samples based upon *CIC* mutation status: wildtype (WT, n = 53), loss of function (LOF, n = 23), and missense mutations (n = 15). LOF mutations were classified as truncating frameshift or stop mutations and missense mutations were only included if they resided within exon 5 (HMG DNA binding domain) or exon 20 (C1 motif). DEA was performed between these three subgroups and identified 564 (WT vs LOF), 420 (WT vs missense), and 23 (LOF vs missense) DE genes (Fold change > 1.5, padj < 0.05; Fig. 2A, Additional file 11: Table S6). Comparison of DE genes between *CIC* LOF and missense mutant samples identified 127 overlapping genes with high directional concordance including known CIC targets (*ETV1, ETV4, ETV5, DUSP4, DUSP6, SPRY4, SHC3*; Fig. 2B). GSEA identified several terms related to regulation of RTK signaling and neural differentiation in both *CIC* LOF and missense tumours (Fig. 2C and D, Additional file 12: Table S7) suggesting that *CIC* missense and LOF mutations result in similar biological consequences on a global transcriptomic level. However, within the 23 DE genes identified between *CIC* LOF and *CIC* missense mutant Type I LGG, *CIC* LOF mutants were found to have increased expression of several genes related to vasculature development and endothelial cell migration (*CD34, HPGD, SRPX2, ANGPT4, DCN, TIMP1*; Additional file 12: Table S7). *CIC* missense mutant Type I LGG also had increased *CIC* expression and decreased *ETV4* expression suggesting that missense mutations may not lead to mRNA transcript decay and are not as fully penetrant as *CIC* LOF mutations [32](Additional file 11: Table S6).

### Curating an in-house cohort of LGG

**Fig. 3:**
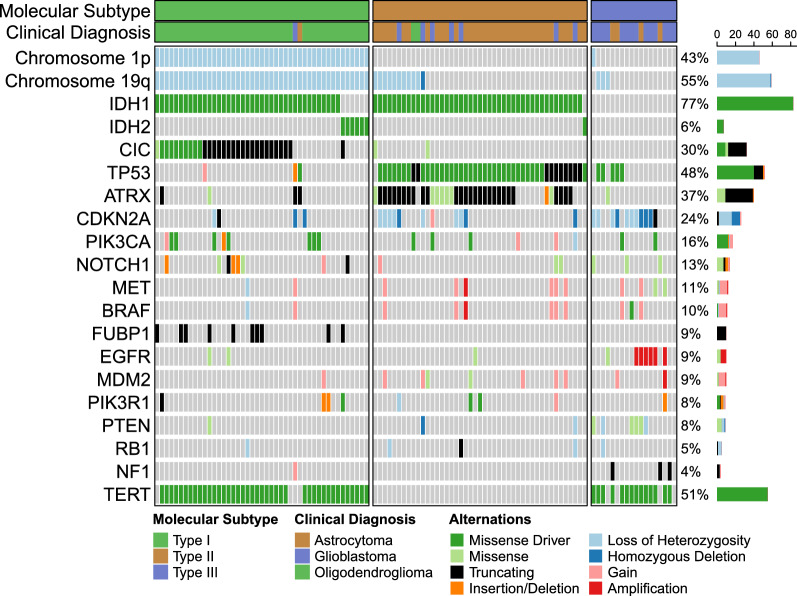
Oncoplot showing mutations identified in our in-house cohort of LGG. Histological and molecular subtypes are displayed above

To begin our proteomic investigation, we curated an in-house cohort of untreated LGG and performed targeted sequencing to determine their molecular subtype. The targeted sequencing panel was designed to target glioma related genes (*IDH1/2, TP53, ATRX, CIC, FUBP1, EGFR, PTEN, CDKN2A/B, NF1, PIK3CA/R1, BRAF, hTERT* promoter) and included probes to determine copy number status of chromosomes 1p/19q, *CDKN2A/B*, *PTEN*, and *EGFR*. A total of 108 samples (57 FFPE and 51 fresh frozen) were collected and DNA extracted. Sequencing identified a total of 45 Type I, 45 Type II, and 18 Type III tumours with some tumours being reclassified compared to their clinical diagnosis, many which were performed prior to the addition of molecular subtyping by the 20WHO (Fig. 3, Additional file 13: Table S8). Within Type I tumours, 30 mutations were identified in *CIC* (20 LOF and 10 missense) which is consistent with previously published studies [4, 5].

### Proteomic signatures are not as pronounce as transcriptomic signatures

**Fig. 4:**
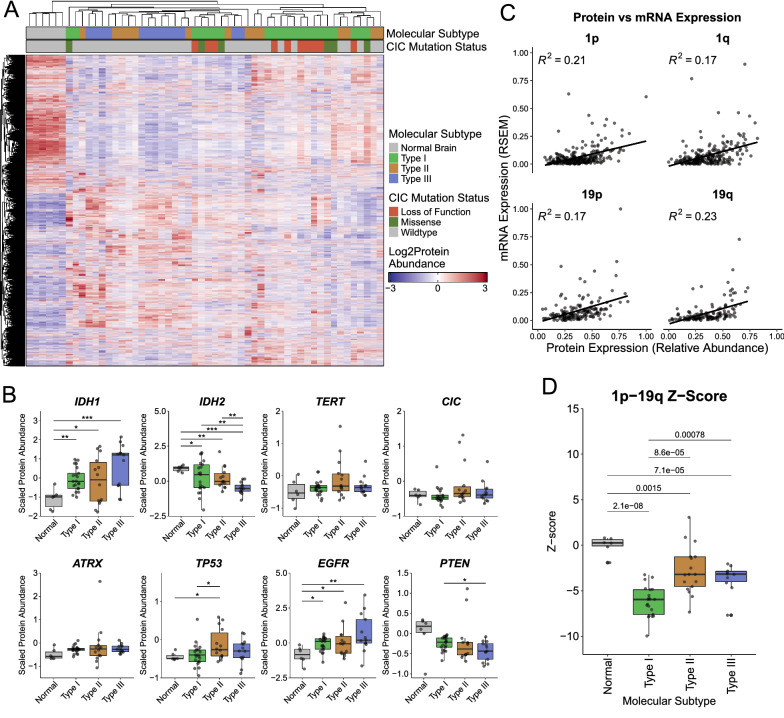
**A** Heatmap and unsupervised clustering of proteins identified in our in-house cohort of LGG. **B** Boxplots comparing the expression of glioma biomarker proteins between normal brain and the 3 LGG subtypes. p-values correspond to * > 0.05, ** > 0.01, *** > 0.001 **C** Scatter plot comparing expression of proteins identified in chromosomes 1p, 1q, 19p, and 19q. Linear regression line is plotted. **D** Boxplot comparing the calculated 1p19q z-score between normal brain and the 3 LGG subtypes

To explore the proteomic differences between the three LGG subgroups, we performed HPLC fractionation followed by tandem mass-tagged mass spectrometry on a cohort of 54 FFPE samples (6 normal brain, 21 Type I, 17 Type II, 10 Type III). Mass spectrometry identified a total of 7988 proteins. After filtering and keeping only proteins identified in all mass spectrometry runs, 5894 proteins remained (Additional file 14: Table S9). Unsupervised clustering using the top 500 variable expressed (median absolute deviation) proteins showed that all 3 LGG subtypes clustered separately from the normal brain controls. However, clustering was variable between the three subtypes (Fig. 4A). Most of the Type I and Type III samples formed two clusters, while Type II samples were dispersed amongst Type I and Type III clusters. Looking at canonical LGG molecular markers, all three LGG subtypes expressed higher levels of IDH1 and EGFR and lower levels of IDH2 compared to normal brain (Fig. 4B). Type III LGG expressed the highest levels of IDH1 which has been shown to contribute to their therapeutic resistance [38]. Type II expressed the highest levels of TP53, consistent with recurrent *TP53* mutations in Type II, and Type III expressed the highest level of EGFR and lowest level of PTEN; again, consistent with recurrent *EGFR* amplification and *PTEN* deletions in Type III. Surprisingly, no differences were detected between subtypes in TERT, ATRX, or CIC; the former two being clinical molecular markers.

Previous protein analysis by TCGA using Reverse Phase Protein Array (RPPA) had identified increased phosphorylated HER3 in Type I, increased expression of SYK, CDH1, and ANXA1 in Type II, and increased expression of HER2 in Type III LGG [3]. Similar to TCGA, we also observed increased expression of SYK and ANXA1 in Type II compared to Type I but levels were similar compared to Type III LGG (Additional file 4: Fig. S4A). Expression of CDH1, HER2, and HER3 were not identified in our cohort and were not looked into. Protein expression of canonically LGG-associated proteins such as TP53, EGFR, and PTEN showed similar trends compared to our cohort (Additional file 4: Fig. S4B and Additional file 15: Table S10).

In our transcriptomic analyses, Type I LGGs were found to be heavily driven by 1p19q co-deletion. To investigate whether this transcriptomic signature translated to the proteome, we compared the mean gene expression and mean protein expression of genes located on chromosomes 1p and 19q within Type I LGG (Fig. 4C). Linear regression between protein and RNA expression found low correlation for chromosome arms 1p (R^2^ = 0.21), 1q (R^2^ = 0.17), 19p (R^2^ = 0.17), and 19q (R^2^ = 0.23). Next, we calculated a combined z-score metric using proteins expressed along chromosomes 1p and 19q compared to normal brain. Using this metric, we found a significant decrease in the expression of 1p19q proteins in Type I compared to Type II and III LGG (Fig. 4D). Interestingly, all three subtypes had significantly decreased expression of 1p and 19q proteins compared to normal brain.

Lastly, we wanted to compare the proteomes between *CIC* mutation status (WT, LOF, missense) within Type I LGG. Concordant with transcriptomic clustering, proteomic clustering did not result in distinct clusters based on *CIC* mutation status or CIC protein expression, (Additional file 3: Fig. S3) and no differences were detected in canonical *CIC* target genes such as ETV4, ETV5, DUSP6, and SPRY4 between *CIC* mutation subgroups (Additional file 3: Fig. S3). Differential protein analysis (DPA) also did not identify any significantly expressed proteins between any of the *CIC* mutation statuses within Type I LGG (Additional file 16: Table S11).

### LGG subtypes can be distinguished using a proteomic classifier

**Fig. 5:**
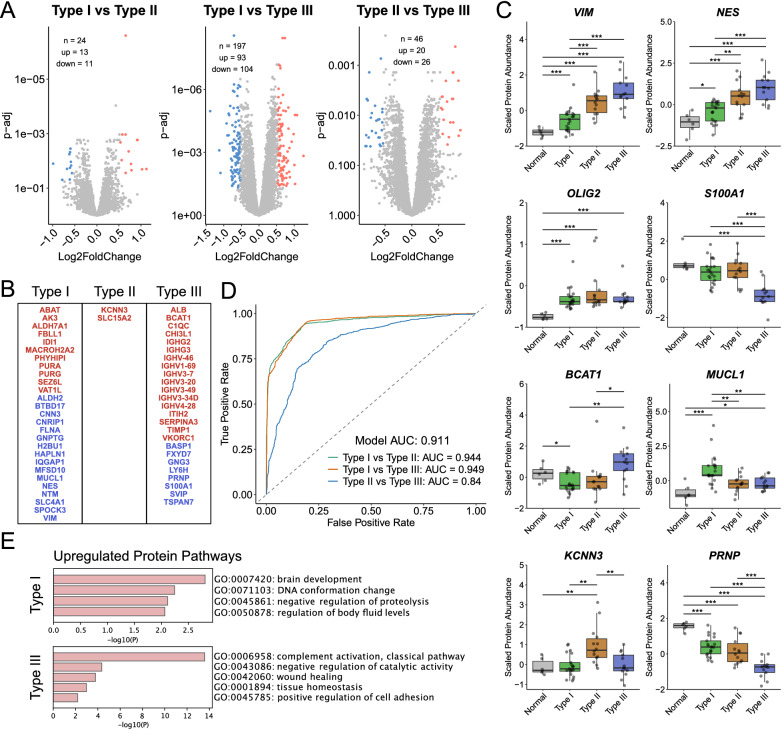
**A** Volcano plots showing differentially expressed proteins identified between Type I, Type II, and Type III LGG. **B** List of subtype specific proteins identified through differential protein analysis. Upregulated proteins are in red and downregulated proteins are in blue. **C** Boxplots comparing the protein expression of glioma and subtype specific proteins between normal brain and the 3 LGG subtypes. p-values correspond to * > 0.05, ** > 0.01, *** > 0.001. **D** AUC-ROC curves for the classification of LGG subtypes based upon the list of subtype specific proteins. Curves was generated using multinomial regression and tenfold cross-validation. **E** Upregulated protein pathways in subtype specific proteins in Type I and Type III LGG

To delve deeper into the proteomic differences between the three LGG subtypes, we performed differential protein expression analysis between the groups (Type I vs Type II, Type I vs Type III, Type II vs Type III). DPA identified 24 (13 up, 11 down) in Type I vs Type II, 197 (93 up, 104 down) in Type I vs Type III, and 46 (20 up, 26 down) in Type II vs Type III, DE proteins (Fold change > 1.5, padj < 0.05; Fig. 5A and Additional file 17: Table S12). Due to the low amount of DE proteins identified between Type I vs Type II and Type II vs Type III, we performed pathway analysis using less stringent parameters (padj < 0.05). Pathway analysis identified increased expression of cell-adhesion proteins in Type I, increased expression of chromatin remodeling proteins in Type II, and increased expression of metabolic pathway proteins in Type III LGG (Additional file 5: Fig. S5 and Additional file 18: Table S13).

Next, to explore potential protein biomarkers for each subtype, we identified proteins that were differentially expressed and directionally concordant between at least two differential comparisons (Type I vs Type II, Type I vs Type III, Type II vs Type III). This resulted in a list of 54 proteins (Fig. 5B): 27 in Type I (11 up, 16 down), 2 in Type II (2 up), and 25 in Type III (17 up, 8 down). Several known glioma markers were identified through this analysis such as Vimentin, Nestin, BCAT1, and S100A1. Depletion of BCAT1 has previously been described as an effective surrogate marker to IDH mutation, consistent with our findings [39]. Expression of Vimentin and Nestin was found to increase stepwise between the three LGG subtypes while others showed increased (BCAT1, MUCL1, KCNN3) or decreased (S100A1, PRNP) expression in only one subtype (Fig. 5C). Using this list of proteins, we trained a classifier using multinomial regression and tenfold cross-validation and found that Type I LGGs were easily distinguishable from both Type II and Type III LGG (AUC = 0.944 and 0.949; Fig. 5D). However, differences between Type II and Type III LGG were not as easily distinguishable (AUC = 0.840) which is consistent with the lack of Type II specific markers identified and the dispersed clustering of Type II LGG (Fig. 4A). Pathway analysis of Type I and Type III specific markers identified upregulation of brain development in Type I and upregulation of immune, inflammation, and wound healing in Type III LGG (Fig. 5E, Additional file 19: Table S14).

## Discussion

Proteomics is one of the fundamental tools in pathology related to identifying protein biomarkers for the differentiation of pathologies and traditionally probed using single protein assays such as immunohistochemistry. The continuing development and maturation of whole proteome studies using mass-spectrometry have opened doors to explore the proteomic landscapes that are innate to specific pathologies and allow for interrogation of pathway and protein susceptibilities on a large scale. While much has been discovered about the genome and transcriptome of low-grade gliomas (LGGs), very few studies have been published investigating the proteomic landscape. Here we present the largest proteo-genomic cohort, to date, of LGG (n = 54) that includes all 3 subtypes (Type I: IDH mutant – 1p19q co-del, Type II: IDH mutant – 1p19q retained, and Type III: IDH wildtype). This cohort is also performed on FFPE suggesting that the challenges associated with fixation can be overcome thus easing the restriction of fresh frozen tissues and increasing the potential cohort size of future proteomic studies.

Transcriptomic analysis of TCGA LGGs revealed, unsurprisingly, strong transcriptomic signatures that were driven heavily by genomic features such as IDH mutation and 1p19q codeletion. Similarly, within Type I LGG, *CIC* mutant (LOF and missense) Type I LGG expressed higher levels of CIC target genes. However, on a global scale, *CIC* mutant Type I LGG did not cluster differentially compared to their *CIC* wildtype counterparts. The lack of differential clustering between the *CIC* WT and mutant Type I LGGs may suggest that there are additional mechanisms that can result in similar global transcriptomic changes, or that *CIC* mutations do not lead to a strong enough global change.

Turning towards proteomic analyses, we found that the distinct transcriptomic signatures we identified using TCGA samples did not translate as strongly into the proteome, resulting in less robust clustering. Type II LGG displayed the most heterogeneity in clustering which may reflect their relation to Type I LGG through IDH mutation but also the propensity of Type II LGG to undergo malignant transformation towards a Type III-like phenotype through modulation of the IDH1 locus [34]. Despite the lack of robust clustering, we were able to identify decreased expression of 1p19q proteins in Type I LGG and a list of 54 subtype specific protein markers. This list surprisingly did not include any molecular markers routinely used in the clinic such as IDH1/2, ATRX, TP53, EGFR, or TERT. In Type I, we identified markers related to brain specific (PURA, ALDH7A2, ABAT) and non-specific (AK3, IDI1, VAT1L) metabolism suggesting there may be metabolic vulnerabilities that can be further explored in Type I LGG [40]. Previously identified vulnerabilities such as upregulation of BCAT1 in IDH wildtype glioma were also identified in our study [41, 42]. Using this list of subtype specific markers, we were able to build a subtype specific classifier using multinomial regression suggesting that a focussed approach is more effective in the proteomic space compared to a global approach in transcriptomics. Similar to the transcriptome, we did not identify any differential clusters or differentially expressed proteins between different *CIC* mutation statuses within Type I LGG. This may be due to post-translational methods of dysregulating CIC that require further exploration [34], and is consistent with previous studies showing CIC protein expression is a better prognostic indicator compared to *CIC* mutation status [36].

## Conclusion

In this study, we explore the transcriptomic and proteomic landscape of LGG and further delve in the transcriptomic and proteomic effects of *CIC* mutation on Type I LGG. Our analyses identify wide-scale transcriptomic signatures driven by IDH mutation and 1p19q-codeletion that are not robustly translated into the proteome. While our proteomic analyses did not result in as robust signatures compared to transcriptomics, we highlight the usefulness of focussed analyses to detect 1p19q codeletion and identify a list of 54 subtype specific protein markers. We show that these 54 protein markers are effective for subtype classification and thus may also be useful for further interrogation and validation. This study also highlights the feasibility of performing global proteomic studies using FFPE tissue and report the largest cohort of LGG to date with molecular subtype information. Future studies could further explore the proteomic landscape between histological grades (2 vs 3 vs 4) and low-grade tumours that undergo malignant transformation into glioblastoma to uncover therapeutic vulnerabilities.

## Supplementary Information

**Additional file1:** Gene set enrichment analysis between the 3 LGG subtypes.

**Additional file 2:** Heatmap and unsupervised clustering of Type I LGG.

**Additional file 3:** Heatmap and unsupervised clustering of proteins within Type I LGG. Lower – Boxplots showing protein expression of CIC and CIC target genes between TypeI LGG CIC subgroups.

**Additional file 4:** Boxplots of protein expression in TCGA proteomics data.

**Additional file 5:** Pathway analysis of differentially expressed proteins between the 3 LGG subtypes.

**Additional file 6:** Patient samples and cohort.

**Additional file 7:** NGS panel design.

**Additional file 8:** Sequencing metrics.

**Additional file 9:** TCGA differential gene expression.

**Additional file 10:** TCGA gene pathway analysis.

**Additional file 11:** Type I differential gene expression.

**Additional file 12:** Type I gene pathway analysis.

**Additional file 13:** Cohort mutations.

**Additional file 14:** Proteins identified.

**Additional file 15:** TCGA Differential protein expression.

**Additional file 16:** Type I differential protein expression.

**Additional file 17:** Differential protein expression analysis.

**Additional file 18:** Protein pathway analysis.

**Additional file 19:** Proteomics classifier pathways.

## Data Availability

Proteomic data is available in the supplemental files. Sequencing FASTQ data has been deposited into the NCBI Sequence Read Archive (accession: PRJNA821547). Data analysis code and figure generation is available at https://github.com/derekwong90/LGG_proteomics.
