# Supplementary figures and images for: Integrated proteomic analysis of low-grade gliomas reveals contributions of 1p-19q co-deletion to oligodendroglioma
Source: Acta Neuropathol Commun. 2022 May 7;10:70. doi: 10.1186/s40478-022-01372-1 (PMC9080204; doi:10.1186/s40478-022-01372-1)

# Upregulated pathways

## Type I (IDH mut, 1p19q co-del)

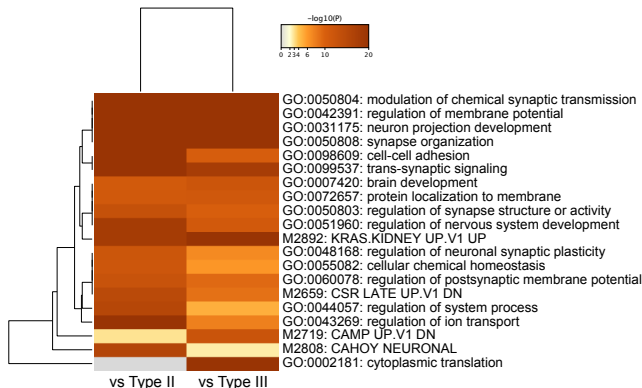

## Type II (IDH mut, 1p19q intact)

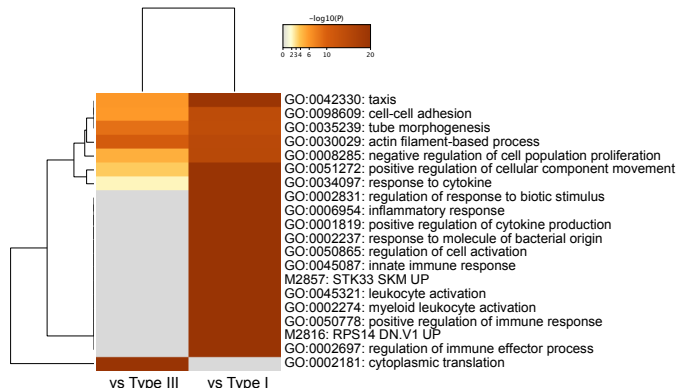

## Type III (IDH WT)

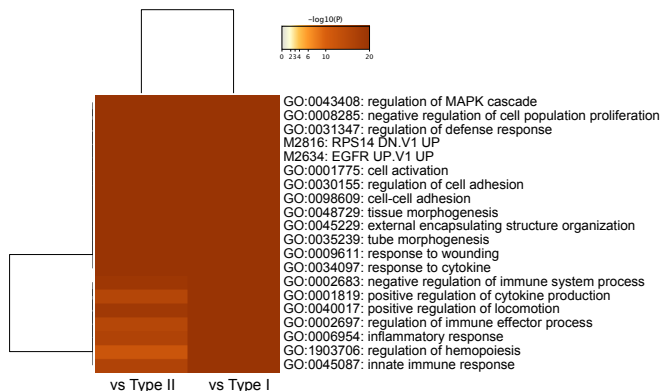

Supplement: Supplementary file 1 — Additional file1: Gene set enrichment analysis between the 3 LGG subtypes. [file 40478_2022_1372_MOESM1_ESM.pdf]

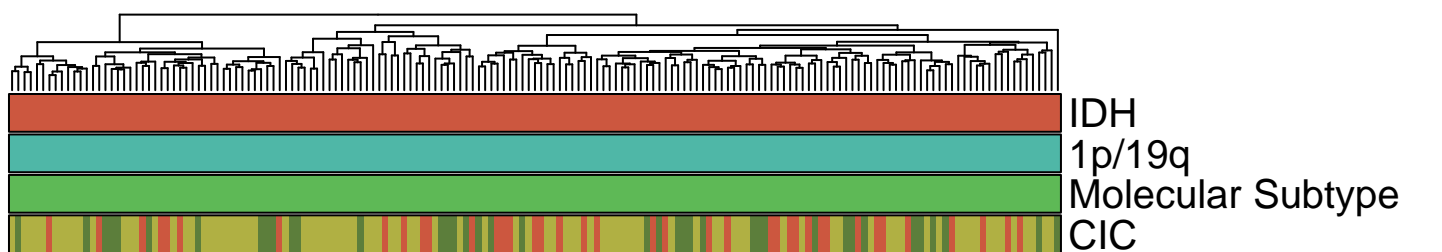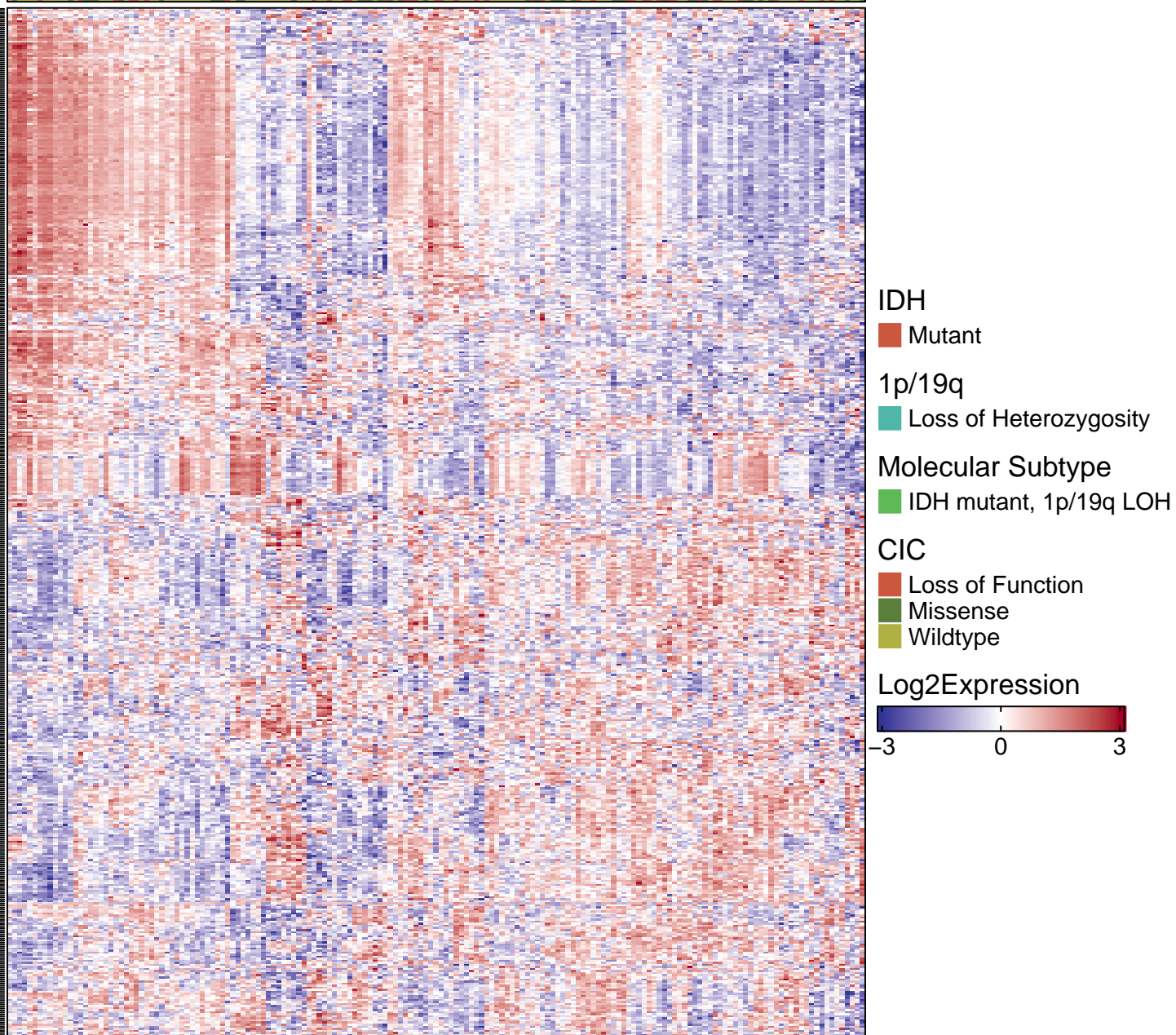

Supplement: Supplementary file 2 — Additional file 2: Heatmap and unsupervised clustering of Type I LGG. [file 40478_2022_1372_MOESM2_ESM.pdf]

A

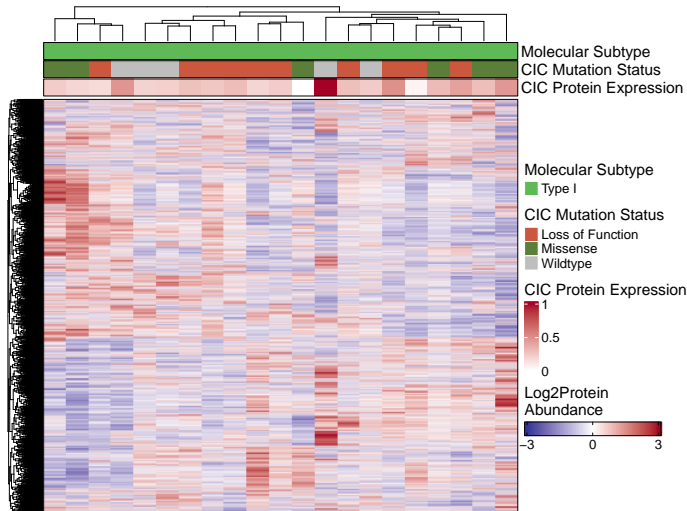

B

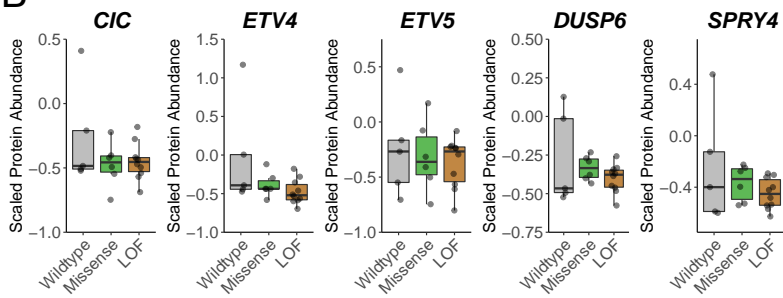

Supplement: Supplementary file 3 — Additional file 3: Heatmap and unsupervised clustering of proteins within Type I LGG. Lower – Boxplots showing protein expression of CIC and CIC target genes between TypeI LGG CIC subgroups. [file 40478_2022_1372_MOESM3_ESM.pdf]

**A**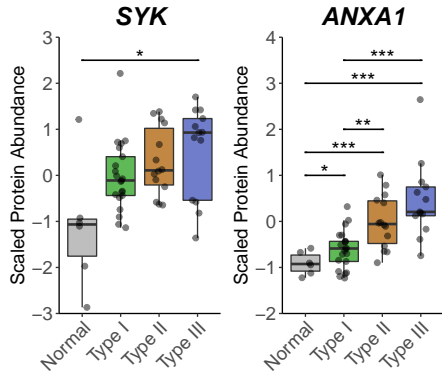**B**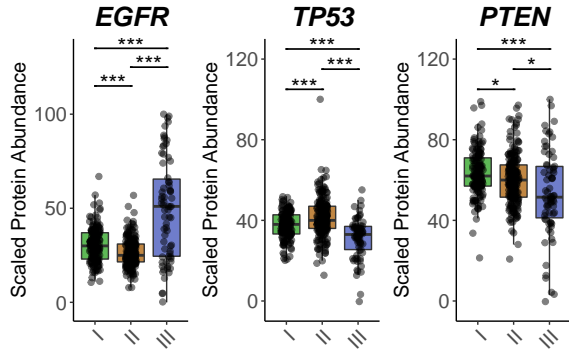

Supplement: Supplementary file 4 — Additional file 4: Boxplots of protein expression in TCGA proteomics data. [file 40478_2022_1372_MOESM4_ESM.pdf]

# Upregulated protein pathways

## Type I (IDH mut, 1p19q code)l)

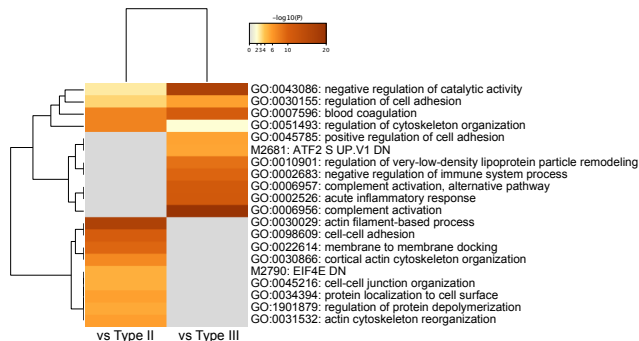

## Type II (IDH mut, 1p19q intact)

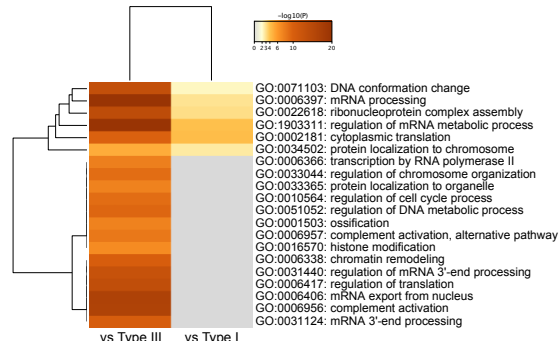

## Type III (IDH WT)

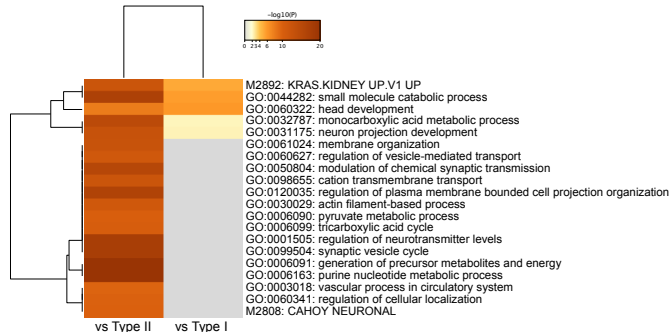

Supplement: Supplementary file 5 — Additional file 5: Pathway analysis of differentially expressed proteins between the 3 LGG subtypes. [file 40478_2022_1372_MOESM5_ESM.pdf]
